# Secular trends of the prevalence of emaciation, overweight, and obesity among school-aged children in Yunnan province, 1985–2019: A serial cross-sectional surveillance study in China

**DOI:** 10.3389/fnut.2022.1037188

**Published:** 2022-12-01

**Authors:** Yunjuan Yang, Jing Dai, Songquan Huang, Tunan Li, Zhizhong Song, Shun Zha, Chengpeng Ma, Litao Chang, Song Zhang, Hong Liu, Diexin Wei, Fan Yang, Limei Dai, Min Tang, Xue Zhang, Yang Liu, Jiming Kang

**Affiliations:** ^1^Health Science Center, Xi'an Jiaotong University, Xi'an, China; ^2^Department of School Health, Yunnan Provincial Center for Disease Control and Prevention, Kunming, Yunnan, China; ^3^Public Health School, Kunming Medical University, Kunming, Yunnan, China; ^4^School of Economics and Management, Kunming University of Science and Technology, Kunming, Yunnan, China; ^5^Department of Hepatology, The Second Affiliated Hospital of Kunming Medical University, Kunming, Yunnan, China; ^6^Bureau of Disease Control and Prevention, Yunnan Health Committee, Kunming, Yunnan, China; ^7^Kunming Primary and Secondary School Health Center, Kunming, Yunnan, China; ^8^Chongqing Three Gorges Medical College, Chongqing, China

**Keywords:** nutrition, overweight and obesity, children, adolescent, epidemic, prevalence, trend

## Abstract

**Objective:**

To understand the trends of nutrition in children and adolescents, which may further help to prevent and control chronic diseases in younger ages.

**Methods:**

The Chinese National Surveys on Students' Constitution and Health (CNSSCH) in Yunnan is a survey of growth conditions, physical fitness, and health status of students in Yunnan and uses a series of complex multistage stratified sampling of seven prefectures consisting of 16 counties. Sampling schools were held constant over 35 years. The participants were randomly selected among 7–18 aged students. We used data from 1985, 1991, 2000, 2005, 2010, 2014, and 2019 CNSSCH of Yunnan. According to body mass index (BMI) criteria of National Working Group for Obesity in China (WGOC-BMI criteria), a participant's nutrition (emaciation, overweight or obesity) was defined. This study is based on survey data from 129,520 participants in 1985 (*n* = 14,683), 1991 (*n* = 4,894), 1995 (*n* = 6,673), 2000 (*n* = 9,751), 2005 (*n* = 23,461), 2010 (*n* = 22,889), 2014 (*n* = 23,003) and 2019 (24,166).

**Results:**

Since 1985, the trends of emaciation over 35 years were decreasing. Regardless of gender, area, and age, the prevalence of obesity and overweight were increased. The average annual growth rate of overweight and obesity was quicker in rural areas and boys than in urban areas and girls. In Yunnan, emaciation, overweight, and obesity disparity in children were common phenomena, with differences in areas and gender.

**Conclusion:**

Children in Yunnan faced the triple burden of malnutrition (emaciation, overweight, and obesity). We should take comprehensive policies and effective intervention measures to decrease the rate of nutrition deficiencies in school-aged children.

## Introduction

Nutrition is associated not only with the growth and physical development of children and adolescents but also with their health throughout their life. A few studies have confirmed that the effects of nutrition on the development of children and adolescents extend to cardiorespiratory fitness, musculoskeletal growth, neurodevelopment, and the immune system, as well as any potential general health of children (1) and increase the risk of noncommunicable diseases in later life (2).

With unprecedented changes in the food environment and lifestyle, the problem of malnutrition is not prominent. Nonetheless, overweight and obesity in childhood and adolescence are the most important global public health challenges and influence all countries in the world (3, 4). The mean age-standardized body mass index (BMI) of children and adolescents has increased globally in most regions from 1975 to 2016. In school-aged children and adolescents, the number of people with obesity has increased by more than 10-fold over the last 40 years, from 11 to 124 million (2016 estimates). This global increase was observed in virtually identical age-standardized mean BMIs from 17.2 kg/m^2^ in 1975 to 18.6 kg/m^2^ for girls in 2016 and from 16.8 to 18.5 kg/m^2^ for boys (5). China is no exception. With rapid economic growth and the urbanization of China, the prevalence of obesity has risen dramatically among children and adolescents in recent decades (4, 6, 7). Some cities (e.g., Beijing, Shanghai, Tianjin, Shijiazhuang, Shenyang, Dalian, Jinan, Qingdao, and Nanjing) had a prevalence of obesity very similar to developed countries (8, 9). Overweight and obesity in childhood not only affect their later growth, physical fitness, and mental health, but also cause obesity in adults, even leading to the early onset of chronic disorders such as hypertension, diabetes, asthma, fatty liver disease, cardiovascular and cerebrovascular diseases, or some advanced tumors (10–15). Obesity in childhood can be a serious threat to adult health (16), which increases the risk of morbidity and mortality in adults (14).

Yunnan province, located in southwest China, is one of the underdeveloped plateau regions with multi-ethnics in China. Few studies have estimated the epidemic situation of nutrition among children and adolescents in Yunnan. This information could guide interventions and strategies that not only prevent children and adolescents from experiencing malnutrition (emaciation, overweight and obesity), but also mitigate its consequences. Since 1985, we have conducted surveys on students' health among school children and adolescents every 5 years in Yunnan. This study aimed to explore the prevalence of nutrition (emaciation, overweight, and obesity) among school-aged children in Yunnan, to estimate the epidemic level and trends of child nutrition in Yunnan province over 35 years. We also provide baseline data for developing child nutrition prevention interventions in Yunnan.

## Materials and methods

### Survey sites

This study was conducted in seven prefectures consisting of 16 counties in Yunnan province, which accounted for 12.4% of the regions in the province, and sampling in schools was maintained over a 35-year period. In total, 62 schools were enrolled in our study with an average of 16,190 (129,520/8) students included annually.

### Data collection

Data were obtained from the Chinese National Surveys on Students' Constitution and Health (CNSSCH) in Yunnan from 1985 to 2019. These surveys were implemented in 1985, 1991, 1995, 2000, 2005, 2010, 2014, and 2019. CNSSCH was a series of surveys with data being collected every 5 years. The sampling method was a complex multistage, cross-sectional, nationwide survey that used a standardized methodology (9).

In brief, at first, the study participants were randomly sampled from the seven surveillance prefectures (namely, Kunming, Honghe, Dali, Lijiang, Lincang, Nujiang, and Xishuangbanna) in Yunnan. In all prefectures, to achieve a better representation within seven prefectures, participants were classified by gender and region (urban or rural) and each of the four groups consisted of equal numbers of individuals from three socioeconomic classes (upper, middle, and lower). In 1985, the five indicators used to define these socioeconomic classes were made prefecture-specific: the regional gross domestic product, total annual income per capita, average food consumption, and regional social welfare index (8, 17, 18). And, the surveillance regions remained constant from 1985 to 2019. Secondly, three urban areas and three rural areas were randomly selected from each prefecture. Then, we randomly selected several schools as surveillance schools in each area (including both junior and senior schools). These schools were randomly selected from a list compiled by the local education committee. And, surveillance schools remained constant from 1985 to 2019. Finally, a list of students from grades 1 to 12 was compiled, and a random selection of two or three classes (depending on their size) was made from each grade. Participants who have diseases in the heart, liver, kidney, and other major organs were excluded. Those who were not willing to sign informed consent were also excluded. Participants aged 7–18 years were randomly selected from surveillance primary and secondary schools in seven prefectures. And, all participants and/or their parents/guardians provided written informed consent. Data were collected through field interviews in all surveys. All surveys used the same sampling methods, surveillance areas, and surveillance schools.

Our study is based on the survey data from 129,520 participants in 1985 (*n* = 14,683), 1991 (*n* = 4,894), 1995 (*n* = 6,673), 2000 (*n* = 9,751), 2005 (*n* = 23,461), 2010 (*n* = 22,889), 2014 (*n* = 23,003), and 2019 (*n* = 24,166). The total number of participants comprises 1.32% of the total number of students in Yunnan.

### Measurements and definitions

The surveys have included a standardized physical examination, which has been conducted by well-trained health workers who followed a reference protocol recommended by the WHO.

Physical examinations included the measurement of individuals' height and weight. Height was measured to the nearest 0.1 cm with a metal column height and sitting height measuring instrument when participants were without shoes. Weight was measured to the nearest 0.10 kg with a balance-beam scale when participants wore lightweight clothing. Both scales and stadiometers were calibrated before use (8, 9, 17, 18). BMI was calculated as weight in kilograms divided by height in meters squared. With reference to WHO Standards, emaciation was defined as BMI <(Means-2SD) (19). Sex- and age-specific BMI cutoff points, recommended by the Working Group for Obesity in China (WGOC-BMI criteria), were used to define overweight and obesity (19). A new BMI classification reference recommended by the WGOC in 2004 is considered to be the most appropriate, shows its superiority in both prospectiveness and authenticity, and is consistent with the Eastern Asia Ethnic characteristics of body fatness growth (20). For both boys and girls aged 7–17 years, overweight was defined as BMI ≥85th percentile but <95th percentile, stratified by gender and age, whereas obesity was defined as BMI ≥95th percentile. For both boys and girls aged 18 years, BMIs of 24.0 and 28.0 kg/m^2^ are set as the cutoffs for overweight and obesity, respectively (19). This study adopted the WGOC-BMI criteria to define overweight and obesity.

### Statistical analysis

The primary data analysis was conducted in 2022. Differences in the mean BMI by age, gender, and area (urban and rural) were compared using ANOVA test. The estimates of the prevalence of emaciation, overweight, and obesity in different survey years were stratified by age, gender, and area. The physical division of different areas was based on the Chinese Administrative Division.

For comparability, the age-standardized and gender-standardized prevalence of the population was calculated using the 2010 China Census as a standard population. Linear by linear association trend and chi-squared analyses were conducted to assess the trends and differences in the prevalence of emaciation, overweight, and obesity for subgroups. All data were analyzed using SPSS 21.0 software. Statistical significance was defined as *p* < 0.05.

## Results

### The demographic characteristics of participants

As shown in Table 1, the study samples were recruited from primary, middle, and high schools in Yunnan, China. A total of 129,520 students were included from 1985, 1991, 1995, 2000, 2005, 2010, 2014, and 2019 surveys, with 64,696 boys and 64,824 girls. The average age was 12.49 ± 3.45 years. In different years, there were no significant differences in the distribution of numbers by gender and age subgroups. These data could be compared (*p* > 0.05). There were 31,463 urban and 98,057 rural participants. There was a significant difference in the distribution of numbers by the area group from 1985 to 2019.

**Table 1:** The distribution of sociodemographic factors in survey children from 1985 to 2019.

**Index**	**1985**	**1991**	**1995**	**2000**	**2005**	**2010**	**2014**	**2019**	***χ^2^***	**P**
**All**	14,683	4,894	6,673	9,751	23,461	22,889	23,003	24,116		
**Gender**									0.16	1.00
Boy	7,342	2,447	3,336	4,879	11,715	11,436	11,494	12,047		
Girl	7,341	2,447	3,337	4,872	11,746	11,453	11,509	12,119		
**Age**									3.73	1.00
7–9	3,672	1,223	1,664	2,447	5,909	5,742	5,749	6,018		
10–12	3,670	1,224	1,659	2,457	5,846	5,754	5,755	6,051		
13–15	3,669	1,224	1,671	2,464	5,824	5,750	5,747	6,062		
16–18	3,672	1,223	1,679	2,383	5,882	5,643	5,752	6,035		
**Area**									15149.70	0.00
Urban	7,341	2,447	4,032	2,521	3,909	3,595	3,600	4,018		
Rural	7,342	2,447	2,641	7,230	19,552	19,294	19,403	20,148		

### Secular changes in BMI

**Figure 1:**
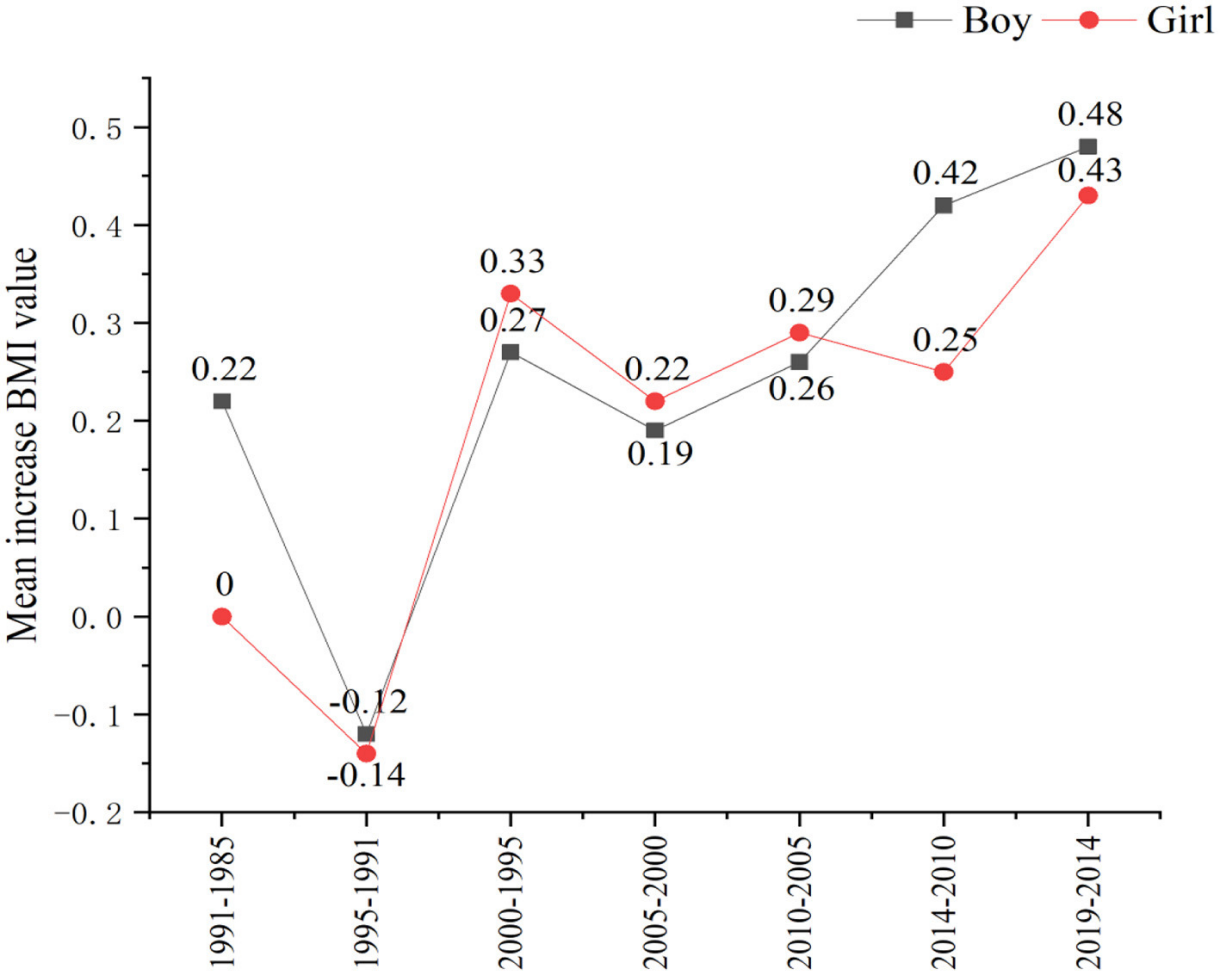
The increase in the mean body mass index (BMI) value of different genders in Yunnan school-aged children from 1985 to 2019.

From 1985 to 2019, the mean BMI of boys increased from 16.82 to 18.54 kg/m^2^, while it increased from 17.14 to 18.52 kg/m^2^ for girls (Table 2). Compared with girls, the mean BMI for boys increased slightly faster. As shown in Figure 1, similar slight decreasing and then increasing trends were seen across all age subgroups and in different genders (*p* < 0.01), with an average annual growth ranging from 0.09% to 0.33% (Table 2). BMI distribution curves for boys shifted upwards between 1985 and 2019, and their upper tails were somewhat elevated. In girls, BMI distribution curves were somewhat volatile.

**Table 2:** The mean BMI of survey children from 1985 to 2019.

**Index**	**1985**	**1991**	**1995**	**2000**	**2005**	**2010**	**2014**	**2019**	***F***	**P**
**All**	16.98 ± 2.62	17.09 ± 2.75	16.96 ± 2.76	17.26 ± 2.86	17.47 ± 2.87	17.74 ± 3.02	18.08 ± 3.23	18.53 ± 3.51	550.98	0.00
**Gender**									53.95	0.00
Boy	16.82 ± 2.34	17.04 ± 2.60	16.92 ± 2.61	17.19 ± 2.78	17.38 ± 2.75	17.64 ± 2.96	18.06 ± 3.26	18.54 ± 3.56		
girl	17.14 ± 2.85	17.14 ± 2.90	17.00 ± 2.90	17.33 ± 2.95	17.55 ± 2.98	17.84 ± 3.06	18.09 ± 3.19	18.52 ± 3.45		
**Age**									25365.79	0.00
7–9	14.55 ± 1.19	14.64 ± 1.50	14.45 ± 1.53	14.89 ± 1.80	15.06 ± 1.78	15.27 ± 1.95	15.54 ± 2.31	15.80 ± 2.39		
10–12	15.65 ± 1.58	15.92 ± 2.07	15.90 ± 2.17	16.06 ± 2.13	16.42 ± 2.33	16.76 ± 2.55	17.35 ± 3.02	17.71 ± 3.23		
13–15	18.06 ± 2.00	18.14 ± 2.13	18.03 ± 2.20	18.29 ± 2.34	18.55 ± 2.45	18.86 ± 2.57	19.11 ± 2.69	19.77 ± 3.07		
16–18	19.67 ± 1.85	19.66 ± 2.02	19.43 ± 1.98	19.87 ± 2.18	19.85 ± 2.15	20.11 ± 2.38	20.30 ± 2.64	20.81 ± 2.97		
**Area**									0.26	0.61
urban	16.80 ± 2.58	17.11 ± 2.79	17.03 ± 2.82	17.92 ± 3.18	17.97 ± 3.27	18.21 ± 3.44	18.56 ± 3.67	18.77 ± 3.75		
rural	17.17 ± 2.64	17.06 ± 2.71	16.86 ± 2.67	17.03 ± 2.71	17.37 ± 2.77	17.65 ± 2.92	17.98 ± 3.13	18.48 ± 3.46		

In some places, there were no statistical differences in mean BMI between urban and rural areas from 1985 to 2019 (*p* > 0.05). However, from 1985 to 2019, the mean BMI in urban areas increased from 16.80 to 18.77 kg/m^2^, while it increased from 17.17 to 18.48 kg/m^2^ in rural areas (Table 2). The mean BMI has a faster rising speed in urban than in rural areas. Differences in the mean BMI between the urban and rural areas increased from 0.37 in 1985 to 0.29 in 2019.

### Secular trends in the prevalence of emaciation

**Figure 2:**
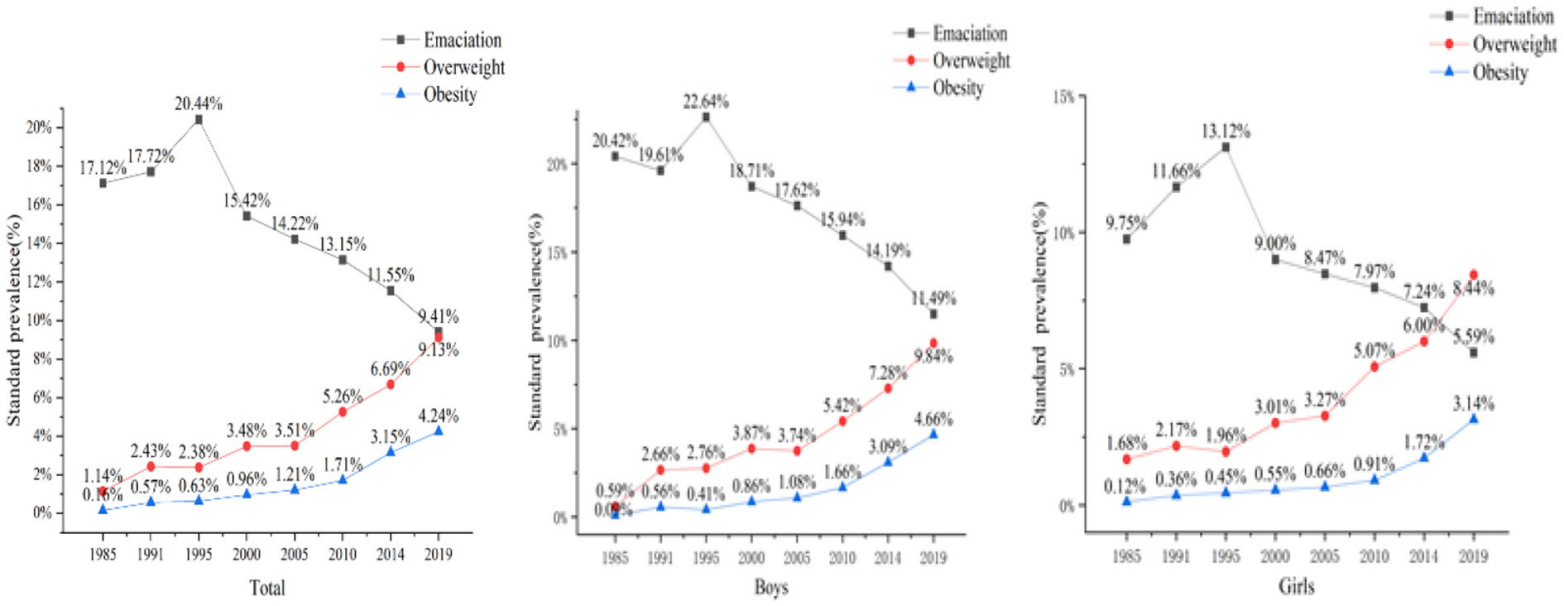
Age-standardized prevalence of emaciation, overweight, and obesity among school-aged children in total and different genders from 1985 to 2019.

The total age-standardized prevalence of emaciation was 13.53%. As shown in Figure 2, in the past 35 years, the trends in age-standardized prevalence of emaciation increased from 1985 to 1995 and decreased significantly year by year after reaching its peak in 1995. The total age-standardized prevalence of emaciation decreased from 20.44 to 9.41% (*p* < 0.05). For boys, the age-standardized prevalence of emaciation decreased from 22.64 to 11.49%. For girls, it decreased from 13.12 to 5.59%. Average annual decreasing rates were 1.39 and 0.94% for boys and girls, respectively. The emaciation rate of boys was still higher than that of girls.

**Figure 3:**
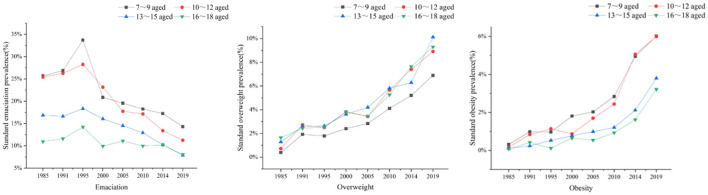
Gender-standardized prevalence of emaciation, overweight, and obesity among school-aged children of different ages from 1985 to 2019.

As age increased, the prevalence of emaciation decreased (Figure 3). This decline was evident in children aged 7–12 years.

In some places, school-aged children in urban areas had a higher prevalence of emaciation than those in rural areas during the same year. Regardless of urban or rural, the prevalence of emaciation decreased over years. In urban areas, the prevalence of emaciation decreased from 23.31% (940/4,032) to 11.27% (453/4,018). Meanwhile, in rural areas, it decreased from 23.48% (620/2,641) to 9.99% (2,012/20,148), with average annual decrease rates of 6.46 and 7.18% in urban and rural, respectively (*p* < 0.01).

### Secular trends in the prevalence of overweight

The total age-standardized prevalence of overweight was 5.17%. Over 35 years, there was a significant increase in the prevalence of overweight among children and adolescents in Yunnan. As shown in Table 3, the total age-standardized prevalence of overweight increased from 1.14 to 9.13% (*p* < 0.05). Age-standardized prevalence of overweight increased from 0.59 to 9.84% and 1.68 to 8.44%, with average annual growth rates of 44.79 and 11.50% for boys and girls, respectively. Trends are shown in Figure 2, and an increase was shown across all age subgroups and in both genders. Obviously, the prevalence of overweight in boys was much higher than that of the same age group in girls, and it increased more quickly in boys.

**Table 3:** The prevalence of overweight and obesity for urban and rural in Yunnan: 1985–2019.

**Survey year**	**Urban**					**Rural**			
	**N**	**Emaciation (%)**	**OV (%)**	**OB (%)**		**N**	**Emaciation (%)**	**OV (%)**	**OB (%)**
1985	7341	1729 (23.55)	63 (0.86)	19 (0.26)		7342	1121 (15.27)	91 (1.24)	6 (0.08)
1991	2447	521 (21.29)	85 (3.47)	20 (0.82)		2447	465 (19.00)	32 (1.31)	10 (0.41)
1995	4032	940 (23.31)	115 (2.85)	34 (0.84)		2641	620 (23.48)	40 (1.51)	12 (0.45)
2000	2521	343 (13.61)	197 (7.81)	62 (2.46)		7230	1342 (18.56)	132 (1.83)	36 (0.50)
2005	3909	575 (14.71)	282 (7.21)	120 (3.07)		19552	3066 (15.68)	531 (2.72)	186 (0.95)
2010	3595	515 (14.33)	350 (9.74)	126 (3.50)		19294	2769 (14.35)	842 (4.36)	294 (1.52)
2014	3600	487 (13.53)	391 (10.86)	213 (5.92)		19403	2402 (12.38)	1121 (5.78)	562 (2.90)
2019	4018	453 (11.27)	443 (11.03)	263 (6.55)		20148	2012 (9.99)	1672 (8.30)	872 (4.33)
All	27445	5563 (20.27)	1483 (5.40)	594 (2.16)		77909	13797 (17.71)	2789 (3.58)	1106 (1.42)

Regardless of the survey year, as age increased, the prevalence of overweight also increased (Figure 3). Children aged 13–15 years increased most obviously.

In some areas, school-aged children in urban areas had a higher prevalence of overweight than those in rural areas during the same year. Regardless of urban or rural, the prevalence of overweight increased. In urban areas, the prevalence of overweight increased from 0.86 to 11.03%. Meanwhile, in rural areas, it rose from 1.24 to 8.30%. Average annual growth rates in urban and rural areas were 33.79% and 16.27%, respectively (*p* < 0.01, Table 3).

### Secular trends in the prevalence of obesity

As shown in Table 3, the total age-standardized prevalence of obesity was 1.91%. The prevalence of obesity also increased significantly over a 35-year period. The total age-standardized prevalence of obesity increased from 0.16 to 4.24% (*p* < 0.05). Age-standardized prevalence of obesity in boys increased from 0.14 to 5.03%, with average annual growth rates of 99.80%. And, for girls, it increased from 0.18 to 3.45%, with average annual growth rates of 51.90%. Disparities between different genders were obvious. In Figure 2, noticeable increases are shown across all age subgroups and in both genders. Obviously, the prevalence of overweight in boys was higher than that of the same age group in girls, and it increased more quickly in boys.

Regardless of the survey year, as age increased, the prevalence rate of obesity also further increased (Figure 3), especially in children aged 10–12 years.

In some regions, school-aged children in urban areas had a higher prevalence of obesity than those in rural areas during the same year. Regardless of urban or rural, the prevalence of obesity increased. For urban areas, the prevalence of obesity increased from 0.26 to 6.55%. For rural areas, it increased from 0.08 to 4.33%, and the average annual growth rates for urban and rural areas were 69.12 and 151.79%, respectively (*p* < 0.01, Table 3).

## Discussion

The age-standardized prevalences of emaciation, overweight, and obesity in the total number of participants were 13.53, 5.17, and 1.91% respectively, among 7–18 school-aged children and adolescents in Yunnan. The main findings of this study were given as follows.

Firstly, the trends in the prevalence of emaciation decreased, while the trends of overweight and obesity increased across gender, age, or areas. The total age-standardized prevalence of overweight increased from 1.14 to 9.13% (*p* < 0.05; the total age-standardized prevalence of obesity increased from 0.16 to 4.24% (*p* < 0.05). The increase in the prevalence of overweight and obesity was more rapid in the most recent decade of 2005–2019 in Yunnan, which is consistent with the findings of a recent Taiwan, Australian, and US study (3, 17, 20–22). This study revealed that the epidemic of emaciation, overweight, and obesity in Yunnan children over 35 years was characterized by the triple burden of nutrition health in Yunnan children, which included the other provinces of China (23). In other words, emaciation was still severe [the rate of emaciation was higher in Yunnan than in China (10.2%)] (23), and secular trends of overweight and obesity among school-aged children and adolescents in Yunnan showed a continuous and fast increase (24–29). This problem may be caused by fast economic development, rising family income (19, 30), and changes in people's lifestyles. The increasing prevalence of overweight and obesity in children and adolescents is bound to increase the risk of chronic diseases, which seriously affects the health status and life quality of children and adolescents in the future.

Secondly, gender disparities in emaciation, overweight, and obesity in children were common phenomena in China (31–35). Yunnan is also no exception. It is mainly manifested in three aspects: first, the mean BMI increased significantly across all age-sex-area-specific subgroups in Yunnan school-aged children since 1985, especially for boys (36–39); second, the age-standardized prevalence of emaciation, overweight, and obesity was higher in boys than in girls; third, the acceleration in the prevalence of overweight and obesity with the average annual growth rate was quicker in boys than in girls (the average annual growth rate of age-standardized prevalence of overweight was 44.79 and 11.50% for boys and girls, respectively; the average annual growth rate of age-standardized prevalence of obesity was 99.80 and 51.90% for boys and girls, respectively). This phenomenon may be related to androgens, which can increase blood pressure by activating the renin-angiotensin system (40). Therefore, boys should be a priority population for the prevention and control of nutrition (including emaciation, overweight, and obesity).

Thirdly, regional differences in the trends and levels of emaciation, overweight, and obesity were evident (32–36, 41–44). This may be due to regional development and differences in individual composition, local economies, and lifestyles. This finding is alarming as the epidemic situation of nutrition among 7–18 school-aged children and adolescents in Yunnan urban areas is accelerating (20, 35, 41–43). Therefore, urban areas should be considered priority areas for the prevention of nutrition.

Fourthly, emaciation among children aged 7–12 years, overweight among children aged 13–15 years, and obesity among children aged 10–12 years all increased significantly. This may be explained by the fact that different populations have different growth patterns. Adolescence is one of the stages of great changes in growth and development. Thus, adolescents should be the priority population for the prevention and control of nutrition.

Based on the results of previous studies, prevention is recognized as the only feasible option to curb the epidemic (36). In the Yunnan survey of CNSSCH in 2014, a multivariate logistic analysis showed that the local average annual temperature and daily physical exercise were the protective factors for protecting children and adolescents from overweight and obesity [odds ratio (OR) values are 0.32 and 0.93, respectively]. Abdominal obesity (OR = 9.53), boys (OR = 1.74), and Han minority (OR = 0.50), living in high latitudes (OR = 2.92) and with a better economic development level (OR = 1.11), playing on the computer or electronic devices every day (OR = 1.14), lack of sleep (OR =1.12) and the heavy burden of conscious work (OR = 1.47) were independent risk factors for overweight and obesity (35, 36, 44, 45). In addition, other studies indicated that different dietary habits (46), different cultures (47), socioeconomic and family economic factors (48), perceptions of body image ideals (49), etc. were factors associated with overweight and obesity. This indicated that we should enhance health education in the prevention and control of nutrition-related health problems, especially taking comprehensive measures to change unhealthy dietary habits, increase the frequency and strength of physical exercise, decrease daily static time of playing on the computer or electronic devices, and getting enough of sleep and relieving the heavy burden of conscious work (50, 51).

### Limitations

This study has some limitations. Firstly, the prevalence of emaciation, overweight, and obesity in 7–18-year-old students may be underestimated. However, this study explored relative changes in its prevalence because, no matter the measures used, the trends were evident. Secondly, this study used data from 35 cross-sectional surveys, with each survey being conducted on different people in field research. It is possible that unintentional errors occurred when estimating the prevalence of emaciation, overweight, and obesity. Lastly, this study did not consider the influence of environmental elements.

### Recommendations

On one hand, in our future research, we should pay more attention to the impact of environmental factors, ethnic culture, and life and behavioral factors. On the other hand, according to a previous study, it was important to develop child-friendly health education materials in the next research. And, we need to concentrate on developing a series of educational electronic animations and pamphlets for children.

## Conclusion

In summary, this study was the first investigation conducted in Yunnan, which used long-term follow-up data and large-scale school-aged children to analyze the trends in emaciation, overweight, and obesity in children and evaluate the epidemic development of nutrition health in children and adolescents in Yunnan. The prevalence of emaciation among 7–18 school-aged children and adolescents in Yunnan decreased from 1985 to 2019. However, the prevalence of emaciation was relatively high in China. The prevalence of overweight and obesity continued to rise in Yunnan. Urban children and adolescents, especially boys, had a higher prevalence of overweight and obesity than other groups. The prevalence of overweight and obesity in school-aged children and adolescents has increased more quickly in the last 5 years than it did in the years before. Thus, nutritional deficiencies, mainly overweight and obesity, have been a major public threat to children and adolescents in Yunnan. Effective comprehensive policies and intervention measures are required to reduce the prevalence of overweight and obesity among school-aged children.

## Data availability statement

The raw data supporting the conclusions of this article will be made available by the authors, without undue reservation.

## Ethics statement

The studies involving human participants were reviewed and approved by the survey of Medical Research Ethics Committee of Yunnan Preventive Medical Institute. Written informed consent to participate in this study was provided by the participants' legal guardian/next of kin.

## Author contributions

YY conceived the study and its design and wrote the manuscript. YY, JD, SH, and TL performed data analysis and altogether drafted the article. ZS, SZha, CM, LC, and SZhan co-ordinated the research sites. YY, HL, DW, FY, LD, and MT performed the survey. XZ and YL performed data analysis and picture drawing. JK tutored and modified the manuscript. All authors contributed to the article and approved the submitted version.

## Funding

This research was supported by the Yunnan Provincial Grant for the Academic Leadership in Medical Sciences (Grant No. D-2018007), the National Natural Science Foundation of China (71764014), the 16th Batch of Kunming Grant for the Young Academic and Technical Leadership (Grant No. KMRCD-2018011), and a grant from the Xishan District Bureau of Science and Technology (Grant No. 34 Xikezi).

## Conflict of interest

The authors declare that the research was conducted in the absence of any commercial or financial relationships that could be construed as a potential conflict of interest.

## Publisher's note

All claims expressed in this article are solely those of the authors and do not necessarily represent those of their affiliated organizations, or those of the publisher, the editors and the reviewers. Any product that may be evaluated in this article, or claim that may be made by its manufacturer, is not guaranteed or endorsed by the publisher.

## Abbreviations

WHO: World Health Organization
BMI: body mass index
CNSSCH: Chinese National Survey on Students' Constitution and Health
WGOC: Working Group for Obesity in China
OV: Overweight
OB: Obesity.
